# Profiling lipidomic changes in dengue-resistant and dengue-susceptible strains of Colombian *Aedes aegypti* after dengue virus challenge

**DOI:** 10.1371/journal.pntd.0011676

**Published:** 2023-10-17

**Authors:** Keenan Elliott, Paola A. Caicedo, Norbert H. Haunerland, Carl Lowenberger

**Affiliations:** 1 Simon Fraser University, Department of Biological Sciences, C2D2 Research Group, Burnaby, British Columbia, Canada; 2 Universidad Icesi, Natural Science Faculty, Department of Biology, Cali, Colombia; WRAIR, UNITED STATES

## Abstract

The mosquito *Aedes aegypti* is the primary vector for all four serotypes of dengue viruses (DENV1-4), which infect millions across the globe each year. Traditional insecticide programs have been transiently effective at minimizing cases; however, insecticide resistance and habitat expansion have caused cases of DENV to surge over the last decade. There is an urgent need to develop novel vector control measures, but these are contingent on a detailed understanding of host-parasite interactions. Here, we have utilized lipidomics to survey the profiles of naturally DENV-resistant (Cali-MIB) or susceptible (Cali-S) populations of *Ae*. *aegypti*, isolated from Cali, Colombia, when fed on blood meals containing DENV. Control insects were fed on a DENV-free blood meal. Midguts were dissected from Cali-MIB and Cali-S females at three time points post-infectious blood meal, 18, 24 and 36h, to identify changes in the lipidome at key times associated with the entry, replication and exit of DENV from midgut cells. We used principal component analysis to visualize broad patterns in lipidomic profiles between the treatment groups, and significance analysis of microarray to determine lipids that were altered in response to viral challenge. These data can be used to identify molecules or metabolic pathways particular to the susceptible or refractory phenotypes, and possibly lead to the generation of stable, DENV-resistant strains of *Ae*. *aegypti*.

## Author summary

Dengue virus (DENV) is a mosquito-transmitted pathogen that can lead to severe complications for infected humans, including hemorrhagic fever or death. Approximately 390 million people are infected with DENV yearly, and reported cases are rising. Currently, we do not have any effective treatments for DENV infection in humans and primarily rely on mosquito control programs to limit transmission. Insecticide-resistant populations, however, are emerging, and there is an urgent need to develop novel methods to control case numbers. We have reported previously on two populations of *Ae*. *aegypti*, the primary vector for DENV, in Cali, Colombia, which are either permissive (Cali-S) or refractory (Cali-MIB) to viral replication. Here we report the use of highly sensitive liquid chromatography-tandem mass spectrometry (LC-MS/MS) to observe changes in the lipidome of the midguts of these strains after challenge with DENV. Using comparative statistics, we identified lipid features significantly altered in the refractory (Cali-MIB) strain after DENV bloodmeal. The compounds identified in this screen could represent molecular targets for future research into developing stable DENV-resistant *Ae*. *aegypti* populations.

## Introduction

Approximately 1.7 billion people globally are at risk of contracting a neglected tropical disease (NTD) [1]. NTDs are a group of infectious diseases that disproportionately affect marginalized individuals in developing nations [1]; among them, dengue virus (DENV) has emerged as one of the most important viral pathogens.

DENV infection causes an acute febrile illness colloquially called *Break-bone fever* for the substantial symptoms associated with infection [2]. Infection with one of four circulating DENV serotypes is often associated with non-life-threatening symptoms that often resolve on their own. In contrast, a subsequent infection with a serotype differing from the primary infection may cause severe dengue or dengue hemorrhagic fever, both of which can be lethal [3]. Approximately half the world’s population, 3.9 billion people throughout 129 countries, live in areas at risk for DENV transmission. Alarmingly, cases reported to the WHO have seen an 8-fold increase in the past two decades [3]. Despite considerable effort, there are currently no effective vaccines or anti-viral treatments recommended by the World Health Organization. The drastic increase in infections and limited pharmaceutical interventions available have raised widespread concern among governments and researchers to uncover novel solutions to reduce viral transmission, infection, and disease.

DENV is transmitted primarily by female *Aedes aegypti* and, to a lesser extent, *Aedes albopictus* [4]. A significant driver of increased DENV case numbers is the expansion of suitable habitats for mosquito vectors [5]. Historically, the most efficacious strategies for mitigating viral spread have relied on vector control measures through insecticides [6], but insecticides may kill non-target and beneficial insects, such as honey bees. In addition, insecticide resistance has developed in many regions due to wide-spread use and overuse. Moreover, insecticide-based vector control measures require continual maintenance. Without proper coordination, insecticide control measures can fail allowing DENV infections to climb. This phenomenon was exemplified during the COVID-19 pandemic; some areas of high-DENV prevalence saw explosions in reported cases due to the absence of vector control programs [7].

More sophisticated molecular vector control measures are gradually coming into use; one prominent example deploys genetically modified insects developed by the biotechnology company Oxitec. In this system, large numbers of male mosquitoes that have been modified to contain a lethal gene are released regularly into an area with high DENV prevalence. These modified males mate with wild-type females, but their offspring never reach adulthood [8]. The result creates a significant depression in mosquito populations in the area where they were released, leading to a decrease in disease transmission. This approach of population suppression requires facilities to raise large numbers of mosquitoes and works best in island settings where immigration of wild type mosquitoes can be controlled. Moreover, the use of genetically modified organisms is often misunderstood and considered controversial by the general public, and therefore gaining approval from local governments can be challenging.

Alternative vector control strategies have modified the mosquito microbiome to reduce or eliminate vector competence [9]. Perhaps the most successful of these programs uses the intracellular bacterium *Wolbachia*. Mosquitoes transinfected with *Wolbachia sp*. experience a considerable decrease in susceptibility to DENV (and other arboviruses) infection and thus transmit DENV at a reduced or zero rate [10]. *Wolbachia-*based vector control programs have been tested with success in the field. However, there is some concern over the circumstances with which *Wolbachia* sp. may block virus replication [11].

Despite the role of *Ae*. *aegypti* as the principal vector for DENV, not all female *Ae*. *aegypti* will transmit DENV. Some females will ingest the virus in a bloodmeal but eliminate DENV before transmitting it. In Cali, Colombia, the proportion of refractory insects (those that do not transmit the virus) in field-collected insects is ~30% [12–14]. The susceptible (Cali-S, susceptible) and the refractory (Cali-MIB, midgut infection barrier) strains are sympatric, and eggs from refractory and susceptible females can be collected within a single oviposition trap. Since their discovery, these susceptible and resistant populations have been reared in the laboratory to study the differences, and to identify those features that determine these phenotypes [12,13,15].

We have studied these selected Cali-S and Cali-MIB strains to understand their physiological differences using suppressive subtractive hybridization assays [12], microarrays [16], RNA-seq [17], RNA interference (RNAi) [16], and microbiome studies [18]. Some of the mechanistic differences between these strains have been elucidated; several differences in immune activation have been observed, including changes in apoptosis regulation and initiation [15]. However, despite considerable effort, we still have an incomplete understanding of the molecular details governing DENV immunity and which factors determine the MIB or S phenotype in these field-derived colonies.

Lipids are intricately involved in the viral life cycle, and previous research has indicated that DENV reprograms the mosquito lipidome to positively influence viral particle production [19–23]. We hypothesized that the Cali-MIB refractory strain may have altered lipid regulation in response to DENV. Specifically, we postulated that changes in the cholesterol and phospholipid metabolism may contribute to the refractory phenotype. Cholesterol has been implicated in numerous studies with *Ae*. *aegypti* infected with DENV [24,25] and has been proposed as a mechanism for DENV resistance in *Wolbachia sp*. control programs [26]. Moreover, changes in phospholipids and phospholipid remodelling have been shown to occur in response to DENV infection in *Ae*. *aegypti*, and altering the balance of lipid precursors impacts this relationship [27,28]. It appears these compounds are important to DENV replication, and may contribute to the refractory phenotype in Cali-MIB insects.

In this study we utilized an untargeted liquid chromatography tandem mass spectrometry (LC-MS/MS) based lipidomics approach to observe changes in the metabolism of lipids in the midgut of Cali-MIB and Cali-S insects in response to DENV challenge. The midgut lipidome was measured at three timepoints following DENV challenge, 18, 24, and 36 hours post blood meal (hpbm), to represent the time when DENV is entering, replicating within, and egressing from the midgut, respectively [13,17,18,29]. Studies based on lipidomics have been utilized previously to ascertain host-virus interaction details in susceptible insects [21,24,25,27]. However, our study system is unique because it allows us to compare the role of lipids in two sympatric populations with a different vectorial competence to DENV, both after the ingestion of a normal blood meal and a bloodmeal containing DENV. This experimental framework is more likely to identify functional differences, as both mosquito strains are subjected to the same metabolic stressor, DENV infection, yet have divergent responses to the virus.

## Materials and methods

### Mosquito rearing

Mosquitoes were maintained under standard laboratory conditions (26 +/- 2°C, 12:12 light-dark cycle with 70% relative humidity). Adults were fed a 10% sugar solution *ad libitum*. Cali-S and Cali-MIB strains were selected as described in [13].

### Virus Isolation and mosquito infection

C6/36 HT cells (*Ae*. *albopictus)* were used to propagate the DENV-2 New Guinea C strain. Cells were infected with DENV-2 and incubated for 14 days at 32°C in L15 media supplemented with 2% heat-inactivated fetal bovine serum, 1% penicillin/streptomycin, and 1% L-glutamine. Virally infected cells were collected in a 15 mL centrifuge tube, and the infected cell suspension was mixed 1:1 with defibrinated rabbit blood to create an infectious blood meal. Infectious blood meal viral titers were quantified using the method described previously [30]. Viral titers in the infectious blood meal ranged from 10^8^ to 10^8.5^ TCID_50_/mL for all oral challenges. Complete details on these procedures have been described previously [13,15].

Five to eight-day-old female adult Cali-S and Cali-MIB *Ae*. *aegypti* females were provided with an infectious blood meal containing DENV for 30 minutes via an artificial pig intestine membrane feeder described previously [13,15]. Control mosquitoes of each phenotype were fed similarly on a blood meal that contained no DENV. After exposure to the blood meals with or without DENV, fully-fed females were transferred to containers and given a 10% sucrose solution ad libitum. Containers had approx. 20 females/container and were maintained under the laboratory conditions described above.

### Mosquito midgut dissections

Midguts from adult female mosquitoes fed on either an infectious DENV blood meal or blood control were dissected on a cold table and immediately transferred to a microcentrifuge tube containing 100% ice-cold methanol at three timepoints; 18, 24, and 36 hours post blood meal (hpbm). Ten midguts were pooled at each timepoint for each treatment to obtain sufficient amounts of tissue for lipid profiling. Each treatment and timepoint was performed in triplicate for both the Cali-S and Cali-MIB strain, for a total of 36 samples. Samples were immediately frozen at -80°C and stored until transportation on dry ice from CIDEIM in Cali, Colombia, to Simon Fraser University in Burnaby, British Columbia. Upon arrival in Burnaby, BC, samples were stored at -80°C.

### LC-MS/MS analysis

Samples were processed and analyzed at The Metabolomics Innovation Centre (TMIC) in Edmonton, Alberta, using an LC-MS/MS global lipidomics approach. Lipids were extracted using a modified Folch liquid-liquid extraction protocol [31]. Aliquots of samples were mixed with NovaMT LipidRep Internal Standard Basic Mix (Nova Medical Testing, Edmonton, Canada), dichloromethane, and methanol. LC-MS was performed in both positive and negative ionization (ESI) for each sample using a Thermo Vanquish UHPLC linked to a Bruker Impact II QTOF Mass Spectrometer. The flow rate was set at 250 μL/min with an m/z range of 150–1500 Da. MS/MS spectra were acquired for all samples with an MS/MS collision Energy of 10–60 eV. Injection volumes were 4 μL and 12 μL for positive and negative ionization, respectively.

## Data processing, feature identification, and data normalization

Data from positive and negative ionization injections were processed separately, and lipid features were aligned using NovaMT LipidScreener (Nova Medical Testing, Edmonton, Canada). Aligned features from positive and negative injections were combined and merged into one intensity table for each sample. Missing values were substituted by one of three methods; 1) for features detected in at least 75% of injections within the group, the median intensity of the sample group was substituted; 2) for features detected in at least 50% of injections, the minimum intensity for features within the group was substituted; 3) for features detected in less than 50% of injections within the group, the global minimum for all samples and QC injections was substituted.

Lipid identification was performed using MS/MS spectral similarity, retention time and accurate mass. A three-tiered approach was used to determine identification confidence; in tier one, MS/MS match score ≥500 and precursor m/z error ≤20.0 ppm and 5.0 mDa: in tier two, MS/MS match score <500 and precursor m/z error ≤20.0 ppm and 5.0 mDa; in tier three, MS match with m/z error ≤20.0 ppm and 5.0 mDa.

Data Normalization was performed using a set of 15 internal standards of differing lipid classes (NovaMT LipidRep Internal Standard Basic Mix). Positive and putatively identified lipids (tiers 1–3) were matched to the internal standards based on similarity to the expected retention times for each lipid class. Intensity Ratios were calculated using the ratio of intensity of each lipid divided by the intensity of the matched internal standard. Unidentified features could not be normalized due to the inability to be matched to an internal standard.

Many duplicate features were identified in the dataset and were removed based on the following criteria. First, the highest tier identified feature was chosen from a set of duplicate features, and the others were discarded. In other words, the order of priority for keeping features was tier 1 > tier 2 > tier 3. If a tie occurred within tier 1 or tier 2 identifications, the feature with the higher MS/MS score was kept. If a tie occurred within tier 3 identifications, the feature with the lowest m/z error was kept.

### Data analysis and visualization

Lipid intensity ratio tables were uploaded to Metaboanalyst 5.0 for analysis and statistical testing [32]. Features with relative standard deviations >25% (SD/mean>25%) in quality control samples (QC) were removed. Samples were normalized by median, and data were scaled with auto-scaling (mean-centred and divided by the SD of each variable). Significance analysis of microarrays (SAM) was used to identify significantly changed features compared with the controls. The delta value for each test was adjusted to control the estimated false discovery rate (FDR) at 5% [33]. Principle Component analysis was performed to assess data quality and between-group differences. Hierarchical clustering was performed using the ward clustering algorithm with Euclidean distance measures. Data visualizations were constructed using a combination of Metaboanalyst 5.0 and the statistical software R [32,34].

## Results

### Control reference frame: Upregulated and downregulated

Throughout this paper, we use the terms *upregulated* and *downregulated* to describe metabolite concentration changes with reference to the comparator (control). For example, if a molecule is decreased in concentration in the susceptible group and there are no changes in concentration in the Cali-MIB strain, this molecule will be identified as upregulated in Cali-MIB when compared to Cali-S. With this reference system, the change in an amount of a measured molecule may be the result of viral propagation in the Cali-S insects and not a condition of viral resistance in the Cali-MIB strain. Nonetheless, the aim of this study is to identify lipids with relative difference between the samples, to provide this information to researchers studying these and other related molecules.

### Feature detection and lipid identification

**Fig 1:**
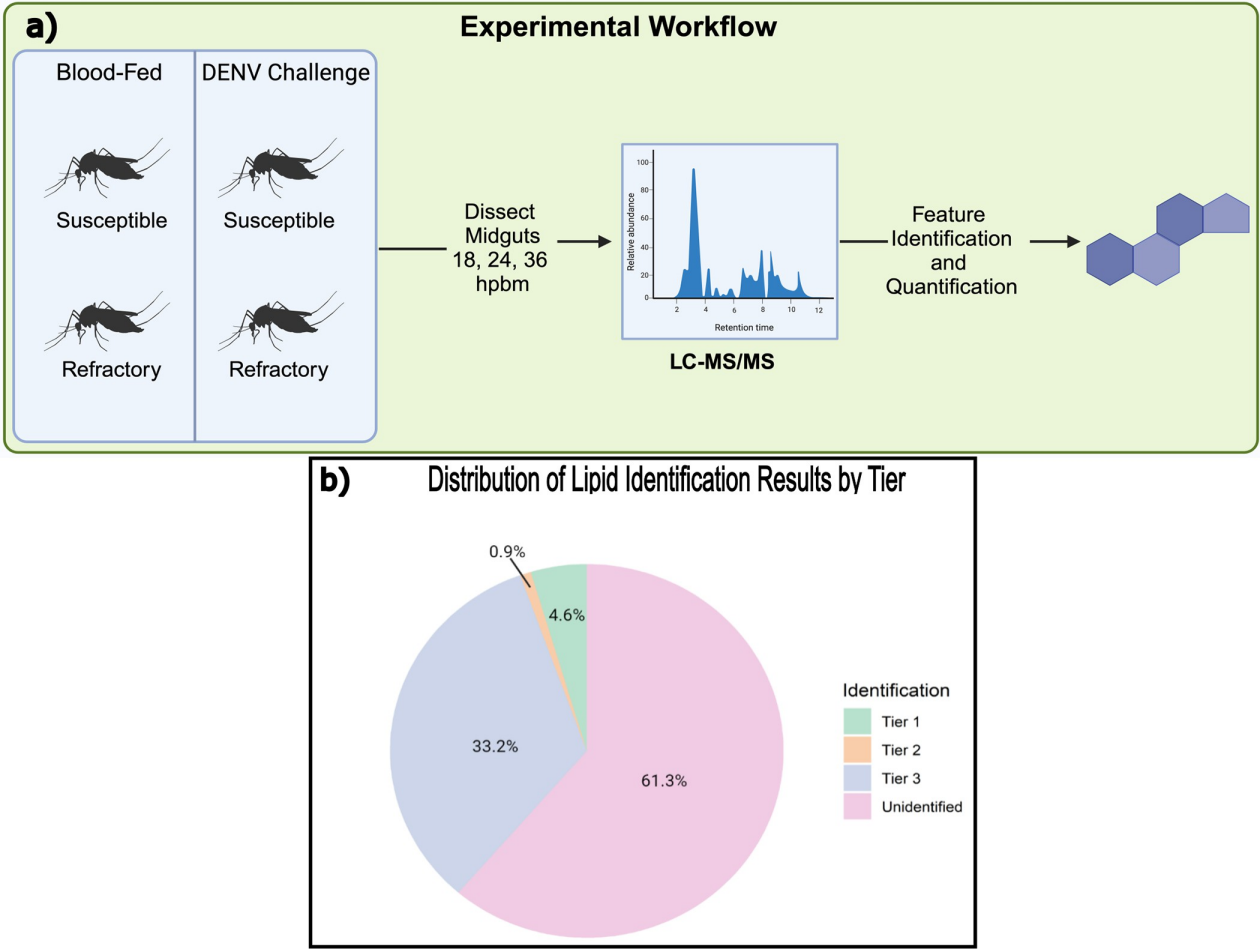
Experimental workflow and distribution of lipid Identifications by tier. **a)** Schematic showing the four treatment groups dissected in triplicate at three time points, 18, 24, and 36 hours post blood meal. Ten midguts were pooled for each treatment at each timepoint and subjected to LC-MS/MS. Created with BioRender.com. **b)** The pie chart shows the percentage of metabolites identified in each accuracy tier.

Lipids have been implicated in the successful entry and replication of DENV in *Aedes aegypti* [21,23–25,27,35]. Accordingly, we evaluated lipid modulations in response to DENV challenge in our system of refractory (Cali-MIB) and susceptible (Cali-S) field-derived *Ae*. *aegypti* strains at time points that are highly relevant to the entry, replication, and escape of DENV in midgut cells; 18, 24 and 36 hours post blood meal (hpbm) (Fig 1).

Three replicates for each treatment and timepoint were extracted for a total of 36 samples. Across all samples, 18749 features were detected. A feature represents a distinct lipid molecule and herein, the terms feature and lipid will be used interchangeably. A three-tier approach was used for feature identification and provided accuracy estimates. If a feature was matched within a tier, it would not be matched again to a subsequent tier. Tiers one and two were matched using accurate mass and MS/MS spectral similarity to provide high-confidence identifications (see Materials and Methods). In tiers one and two, 863 (4.6%) and 169 (0.9%) features were identified, respectively (Fig 1). Tier three uses mass-matching to provide putative lipid identifications. Within this tier, 6223 (33.2%) features were identified (Fig 1). Two analyses were performed using these data, one containing all identified features (tiers 1, 2, and 3) and one using only high confidence metabolites (tiers 1 and 2).

### Principal component analysis: Tiers 1, 2, and 3

Principal component analysis (PCA) was applied to the full dataset to assess data trends and data quality. PCA is a widely used form of data compression and aims to model high-dimensional data in a lower-dimensional space while preserving most of the variance within the data [36]. We performed three comparative PCAs at each timepoint: Cali-S females fed on blood containing virus (Cali-S+DENV) vs. Cali-S females fed only on blood (Cali-S+B), Cali-MIB females fed on blood containing virus (Cali-MIB+DENV) vs. Cali-MIB females fed only on blood (Cali-MIB+B), and Cali-MIB+DENV vs. Cali-S+DENV. The third analysis compares the lipid profiles in each strain when they were both challenged with DENV and is the focus of the present study. Because both strains are under the same metabolic stressor, DENV, this comparison has the highest potential to provide meaningful compounds contributing to their respective phenotypes.

**Fig 2:**
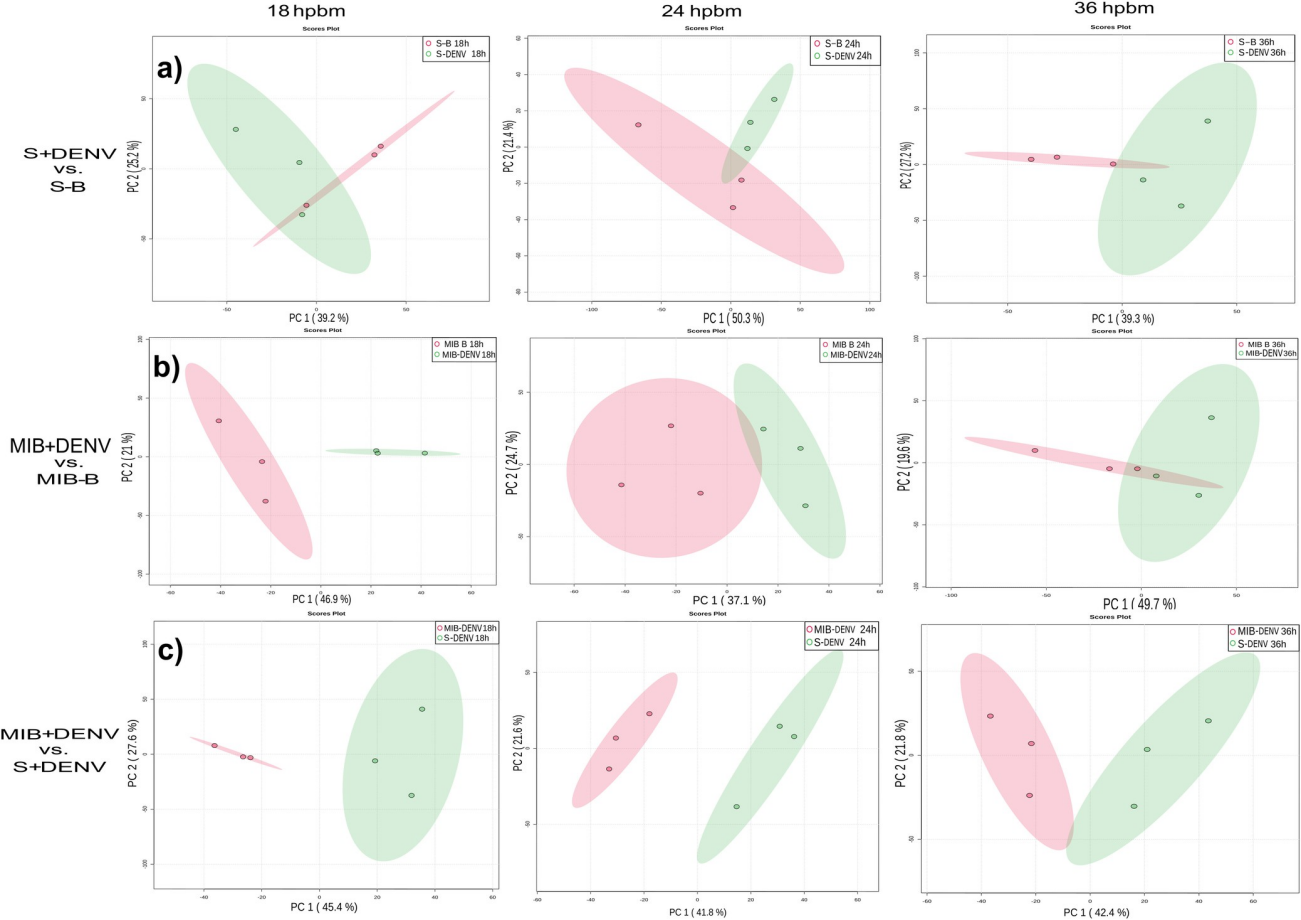
PCA plots of all comparisons from Tier 1, 2, and 3 data. Principal component analysis (PCA) of a) Cali-S+DENV vs. Cali-S+B, b) Cali-MIB+DENV vs. Cali-MIB+B, and c) Cali-MIB+DENV vs. Cali-S-DENV at 18, 24, and 36 hpbm. The X-axis has the first principal component and the Y-axis the second. Each data point represents one sample of 10 pooled insect midguts with the 95% confidence interval displayed as the ellipsis. In a) and b) virus-challenged samples are shown in green, and blood-fed only samples are shown in red. In c) Cali-MIB samples are in red, and Cali-S samples are in green.

PCA plots for all timepoints and treatments can be viewed in Fig 2. Cali-S+DENV samples show modest separation compared with Cali-S+B controls for all timepoints (Fig 2A). At 18 hpbm, the Cali-S+DENV overlaps with Cali-S+B controls. The PCA plot of 24 hpbm shows increased separation, yet the 95% confidence intervals (coloured ellipses) overlap, and this trend holds at 36 hpbm. Interestingly, the plots of Cali-MIB samples show an inverse trend. At 18 hpbm, the greatest separation is observed with strong inter-group differences, which is reduced at 24 hpbm and 36 hpbm (Fig 2B). PCA plots from the comparison of Cali-MIB+DENV and Cali-S+DENV samples show strong separation at all three timepoints (Fig 2C), indicating there may be significant differences in the lipid profile when responding to DENV.

### Principal component analysis: Tiers 1 and 2

**Fig 3:**
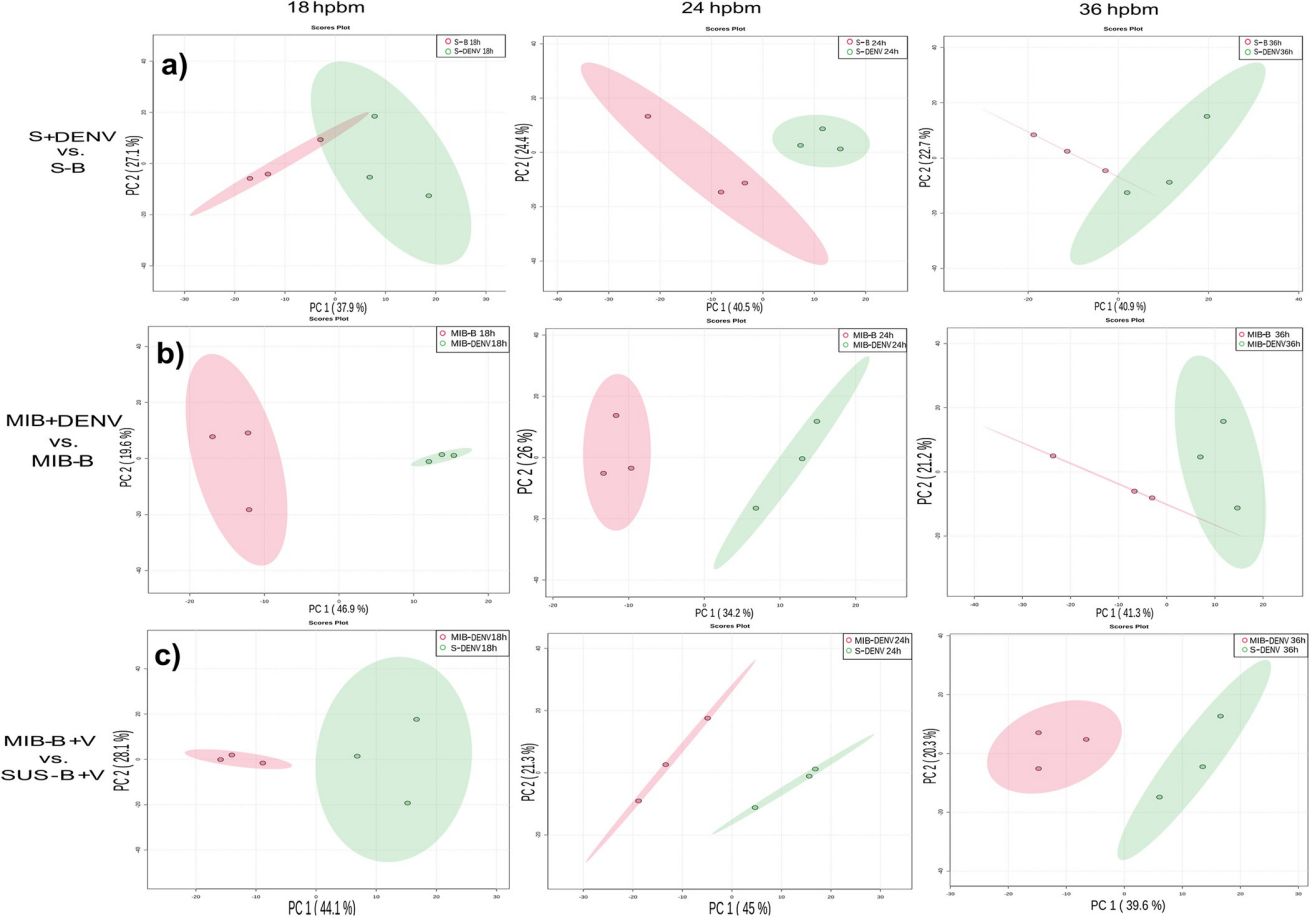
Principal component analysis (PCA) plots of all comparisons from Tier 1 and 2 data. PCA of a) Cali-S+DENV vs. Cali-S+B, b) Cali-MIB+DENV vs. Cali-MIB+B, and c) Cali-MIB+DENV vs. Cali-S+DENV, at 18, 24, and 36 hpbm. The X-axis has the first principal component, and the Y-axis the second. Each data point represents one sample of 10 pooled insect midguts with the 95% confidence interval displayed as the ellipsis. In a) and b) virus-challenged samples are shown in green, and blood-fed only samples are shown in red. In c) Cali-MIB samples are red, and Cali-S samples are green.

Our dataset was subdivided into high-confidence lipid identifications and putative lipid identifications. We evaluated the dataset’s quality with PCA using only the high-confidence lipids. PCA plots for the Cali-S+DENV compared with Cali-S+B controls showed similar trends to the full feature set (tiers 1, 2, and 3). At 18 hpbm, there is modest separation between treatment and control (Fig 3A). By 24 and 36 hpbm, the two groups are distinguishable by PCA.

Observing the PCA plots for the Cali-MIB+DENV samples compared with Cali-MIB+B shows separation at 18 and 24 hpbm, which is reduced by 36 hpbm (Fig 3B). PCA plots from Cali-S and Cali-MIB samples using the high-confidence features trend similarly to plots using the full feature set. Interestingly, there is a greater resolution when using the reduced feature set.

PCA plots from Cali-MIB+DENV samples compared with Cali-S+DENV samples show strong separation at all three timepoints (Fig 3C). None of the plots have overlapping confidence bands. At 24 hpbm, the narrowest confidence band and the greatest separation can be observed. The results from PCA analysis show that there are likely differences in the lipid profiles of Cali-MIB and Cali-S insects while responding to DENV challenge.

### Identifying significant lipids and lipid categories- Tiers 1, 2, and 3

After observing trends across treatment groups using PCA, we determined lipid categories and lipid features that were altered in each of the comparisons. Two statistical methods were considered to determine significant features, significance analysis of microarray (SAM) and partial least squares-discriminant analysis (PLS-DA). The features identified as significant by these methods showed considerable overlap. Ultimately SAM was chosen to select significantly altered lipids because the algorithm also provides an estimated false discovery rate (FDR) [33]. For all tests, the delta value was adjusted to control the estimated FDR at 5%.

**Fig 4:**
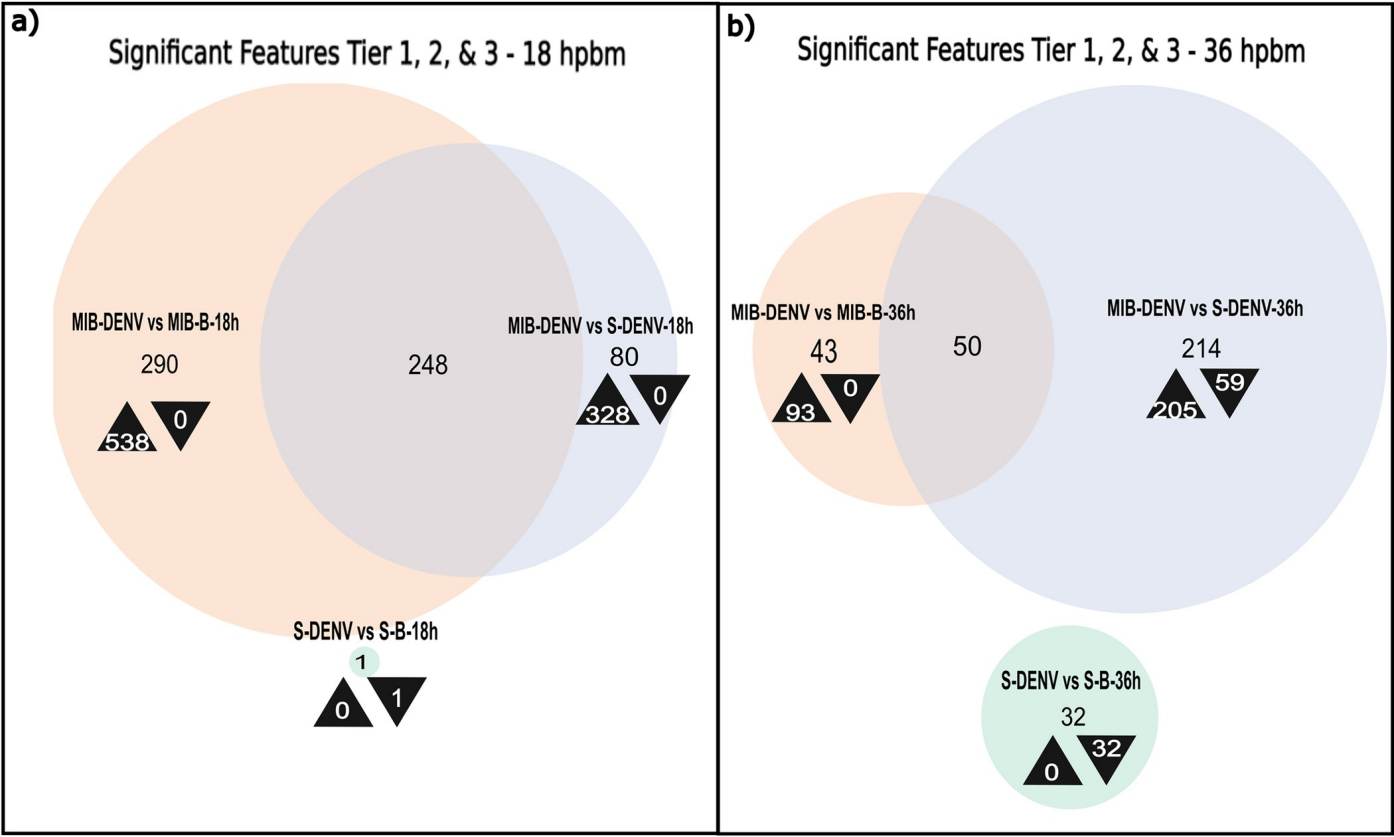
Venn Diagram of significant features, Tier 1, 2, and 3–18, 36 hpbm. Venn Diagrams showing the number of significantly differently regulated lipids selected using the SAM model at each time point, for each sample comparison (Cali-S+DENV vs. Cali-S+B, Cali-MIB+DENV vs. Cali-MIB+B, Cali-MIB+DENV vs. Cali-S+DENV). Black arrowheads pointing up depict the number of metabolites that were increased in concentration compared to the control group, and arrowheads pointing down show the number of metabolites that were decreased in concentration compared to the control group. The control groups are listed second in each comparison.

The lipids identified to be the most significantly different for each time-point and treatment can be seen in Table 1. When comparing Cali-S+DENV with Cali-S+B, only one lipid was found to be significantly different at 18 hpbm (Fig 4A). This contrasts with the Cali-MIB strain that showed 538 differently regulated lipids at the same timepoint compared with non-infected controls (Cali-MIB+DENV vs Cali-MIB+B). Cali-MIB+DENV, when compared with Cali-S+DENV, had 328 significant lipids (Fig 4A). Moreover, there is considerable overlap of the 538 lipids altered in Cali-MIB within-group comparisons and the 328 significant lipids in the Cali-MIB vs Cali-S comparison. The Venn diagram shows that 248 lipids were identified as significant in both groups (Fig 4A). Additionally, there were noticeable patterns of lipid regulation among the various lipid profiles. The single lipid deemed significant in the Cali-S+DENV vs Cali-S+B comparison was downregulated (decreased in concentration relative to the control), whereas in the other two comparisons (Cali-MIB+DENV vs Cali-MIB+B and Cali-MIB+DENV vs Cali-S+DENV) all lipids were upregulated (increased in concentration relative to controls) (Fig 4A).

**Table 1:** Top 10 significantly identified features in tiers 1, 2, and 3. Significance was determined using SAM modelling, with an empirical FDR at 5%. If less than ten features were significant at the 5% threshold, then only those features are listed. Features are listed in alphabetical order, and those appearing in more than one comparison are listed in bold typeface.

18 HPBM		
Cali-S-DENV vs Cali-S-B 18hpbm	Cali-MIB-DENV vs Cali-MIB-B 18hpbm	Cali-MIB-DENV vs Cali-S-DENV 18hpbm
PC O-20:2-DR	PA O-44:6-UR	ACer 62:0;O3-UR
	Cer 38:0;O3-UR	**Cer d16:0_12:0;O2-UR**
	**Cer d16:0_12:0;O2-UR**	**Cer d18:0_22:0;O2-UR**
	**Cer d18:0_22:0;O2-UR**	**Cer d18:0_22:1;O2-UR**
	**Cer d18:0_22:1;O2-UR**	**Cer d18:1_18:1;O3-UR**
	**Cer d18:1_18:1;O3-UR**	**Cer d22:0_16:0;O2-UR**
	**Cer d22:0_16:0;O2-UR**	**FA 14:0;O-UR**
	**FA 14:0;O-UR**	HexCer 36:0;O5-UR
	PG 30:8-UR	PR 32:0;O-UR
	**PR 40:15;O4-UR**	**PR 40:15;O4-UR**
**24 HPBM**		
Cali-S-DENV vs Cali-S-B 24hpbm	Cali-MIB-DENV vs Cali-MIB-B 24hpbm	Cali-MIB-DENV vs Cali-S-DENV 24hpbm
		FA 18:0-DR
		FA 22:0;O4-DR
		PA O-39:1-DR
		PC 14:1_24:2-DR
		PC 16:2_24:4-DR
		PC 18:1_18:2-DR
		SHexCer 44:0;O2-DR
		ST 20:3;O4;GlcA-DR
		TG 12:0_12:1_18:5-DR
		TG 18:1_18:2_18:3-DR
**36 HPBM**		
Cali-S-DENV vs Cali-S-B 36hpbm	Cali-MIB-DENV vs Cali-MIB-B 36hpbm	Cali-MIB-DENV vs Cali-S-DENV 36hpbm
ACer 92:4;O5-DR	Cer 35:2;O3-UR	3-hydroxy-3-methyl-5-oxo-UR*
Cer 35:1;O4-DR	FA 38:6-UR	Car 24:0;O-UR
Hex2Cer 28:0;O6-DR	FA 43:0;O-UR	Car 25:0;O2-DR
Hex2Cer 32:5;O4-DR	Hex2Cer 30:0;O6-UR	Cer 34:0;O3-UR
Hex2Cer 43:3;O4-DR	PE-Cer 34:4;O2-UR	DGCC 36:5-UR
LPI O-32:6-DR	PE-Cer 36:4;O2-UR	Leupeptin-DR
PC 16:1_18:1-DR	PG O-37:0;O-UR	MIPC 30:2;O3-UR
PC 18:2_18:2-DR	PI O-40:0;O-UR	PC 26:3;O3-DR
PIP3 36:9-DR	SM d14:0_13:0-UR	PI-Cer 37:6;O6-UR
SHexCer 37:2;O2-DR	SM d14:0_15:0-UR	PR 20:0;O2 -UR

* = 3-hydroxy-3-methyl-5-oxo-5-[[(2R,3S,4S,5R,6S)-3,4,5-trihydroxy-6-(2-methyl-4-oxopyran-3-yl)oxyoxan-2-yl]methoxy]pentanoic acid.

Within the full dataset, no lipid features were significantly different at 24 hpbm in either Cali-S or Cali-MIB insects challenged with DENV compared with their blood-fed controls (Table 1). However, Cali-MIB+DENV compared with Cali-S+DENV showed 38 significantly different lipids, all with reduced amounts in the Cali-MIB samples.

At 36 hpbm, more lipids were identified as significant than at the 24 hpbm time point; 32, 93 and 264 features were identified as significant in Cali-S+DENV vs Cali-S+B control, Cali-MIB+DENV vs Cali-MIB+B controls, and Cali-MIB+DENV vs Cali-S+DENV comparisons, respectively (Fig 4B). Significant lipids in Cali-S+DENV vs Cali-S+B samples were all downregulated, whereas in Cali-MIB+DENV vs Cali-MIB+B samples, all lipids were upregulated. Cali-MIB+DENV vs Cali-S+DENV samples had a more balanced regulatory pattern, with 205 lipids upregulated and 59 downregulated. The two comparisons using Cali-MIB samples also showed considerable overlap with 50 common lipids between the two comparisons (Fig 4B).

**Fig 5:**
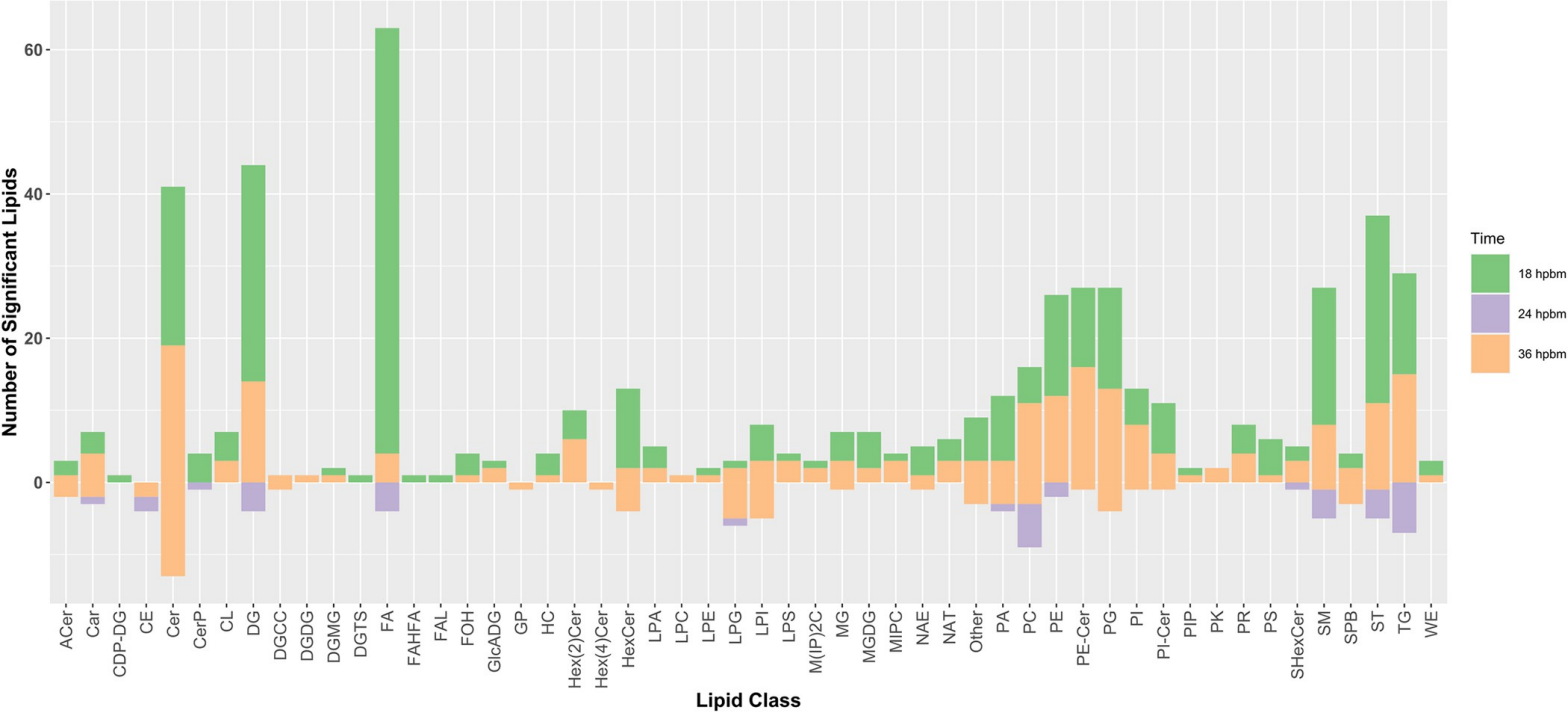
Significant lipids by category-Tiers 1, 2, and 3. Number of significant lipids regulated in the Cali-MIB+DENV vs Cali-S+DENV comparison, by lipid category. Each lipid category is divided by timepoint, with 18 hpbm shown in green, 24 hpbm shown in purple, and 36 hpbm shown in orange. The number of significant lipids differently regulated is shown on the Y-axis, with positive values indicating an increase in concentration compared with Cali-S+DENV samples, and negative values indicating a decrease in concentration. Abbreviations for lipid categories shown on the X-axis can be seen in Table 2.

The lipidome is in constant flux with nearly infinite numbers of possible compounds. Therefore, it can be informative to observe changes in categories of lipids rather than observe alterations in individual lipid features. Lipids were placed into one of 72 different lipid subclasses based on their identification within the NovaMT LipidScreener platform. Significant features in the Cali-MIB+DENV vs Cali-S+DENV comparison were sorted by their lipid categorization and plotted to identify the most significant categories at each timepoint (Fig 5). At 18 hpbm, the top lipid categories among significant features are fatty acids (FA), diacylglycerols (DG), ceramides (Cer), and sterol lipids (ST) (Fig 5). At 24 hpbm fewer features were significant. Nonetheless, the largest lipid categories were triacylglycerols and Phosphatidylcholines (Fig 5). At 36 hpbm, the changes in lipid categories were broader, with ceramides having the greatest number of significant lipids (Fig 5). A full list of lipid categories and abbreviations used in Fig 5 can be seen in Table 2.

**Table 2:** List of lipid categories and abbreviations.

Classification (Category–Main Class–Subclass)	Abbreviation
Sphingolipids—Ceramides—Acylceramides	ACer
Fatty Acyls—Fatty esters—Fatty acyl carnitines	Car
Glycerophospholipids—Cytidine-5’-diphosphate (CDP)-Glycerols—CDP-diacylglycerols	CDP-DG
Sterol Lipids—Sterols—Steryl esters	CE
Sphingolipids—Ceramides—N-acylsphingosines (ceramides)	Cer
Sphingolipids—Ceramides—Ceramide 1-phosphates	CerP
Glycerophospholipids—Glycerophosphoglycerophosphoglycerols—Monoacylglycerophosphoglycerophosphomonoradylglycerols (Cardiolipins)	CL
Glycerolipids—Diradylglycerols—Diacylglycerols	DG
Glycerolipids—Other Glycerolipids (hydroxymethyl-choline)	DGCC
Glycerolipids—Glycosyldiradylglycerols–Glycosyldiacylglycerols (Digalactosyldiacylgylcerols)	DGDG
Glycerolipids—Glycosylmonoradylglycerols–Glycosylmonoacylglycerols (Digalactosylmonoacylgylcerols)	DGMG
Glycerolipids—Other Glycerolipids (trimethyl-homoserine)	DGTS
Fatty Acyls—Fatty Acids and Conjugates—Fatty acids	FA
Fatty Acyls—Fatty esters—Fatty acid estolides	FAHFA
Fatty Acyls—Fatty aldehydes	FAL
Fatty Acyls—Fatty alcohols	FOH
Glycerolipids—Glycosyldiradylglycerols—Glycosyldiacylglycerols	GlcADG
Glycerophospholipids—Other Glycerophospholipids	GP
Fatty Acyls—Hydrocarbons	HC
Sphingolipids—Glycosphingolipids—Hexosyl ceramides	HexCer
Sphingolipids—Glycosphingolipids—Hexosyl ceramides (2 hexosyl groups)	Hex[2]Cer
Sphingolipids—Glycosphingolipids—Hexosyl ceramides (4 hexosyl groups)	Hex[4]Cer
Glycerophospholipids—Glycerophosphates—Monoacylglycerophosphates (lysophosphatidic acids)	LPA
Glycerophospholipids—Glycerophosphocholines—Monoacylglycerophosphocholines (lysophosphatidylcholines)	LPC
Glycerophospholipids—Glycerophosphoethanolamines—Monoacylglycerophosphoethanolamines (lysophosphatidylethanolamines)	LPE
Glycerophospholipids—Glycerophosphoglycerols—Monoacylglycerophosphoglycerol (lysophosphatidylglycerol)	LPG
Glycerophospholipids—Glycerophosphoinositols—Monoacylglycerophosphoinositols (lysophosphatidylinositols)	LPI
Glycerophospholipids—Glycerophosphoserines—Monoacylglycerophosphoserines (lysophosphatidylserines)	LPS
Sphingolipids—Phosphosphingolipids—Ceramide phosphoinositols	M(IP)2C
Glycerolipids—Monoradylglycerols—Monoacylglycerols	MG
Glycerolipids—Glycosyldiradylglycerols–Glycosyldiacylglycerols (Monogalactosyldiacylgylcerols)	MGDG
Sphingolipids—Phosphosphingolipids—Ceramide phosphoinositols	MIPC
Fatty Acyls—Fatty amides—N-acyl ethanolamines (endocannabinoids)	NAE
Fatty Acyls—Fatty amides—N-acyl amines (taurines)	NAT
Glycerophospholipids—Glycerophosphates–Diacylglycerophosphates (Phosphatidic Acids)	PA
Glycerophospholipids—Glycerophosphocholines–Diacylglycerophosphocholines (Phosphatidylcholines)	PC
Glycerophospholipids—Glycerophosphoethanolamines–Diacylglycerophosphoethanolamines (Phosphatidylethanolamines)	PE
Sphingolipids—Phosphosphingolipids—Ceramide phosphoethanolamines	PE-Cer
Glycerophospholipids—Glycerophosphoglycerols—Monoacylglycerophosphoglycerols (Phosphatidylglycerols)	PG
Glycerophospholipids—Glycerophosphoinositols–Diacylglycerophosphoinositols (Phosphatidylinositols)	PI
Sphingolipids—Phosphosphingolipids—Ceramide phosphoinositols	PI-Cer
Glycerophospholipids—Glycerophosphoinositol monophosphates	PIP
Polyketides	PK
Prenol Lipids	PR
Glycerophospholipids—Glycerophosphoserines–Diacylglycerophosphoserines (Phosphatidylserines)	PS
Sphingolipids—Acidic glycosphingolipids—Sulfoglycosphingolipids (sulfatides)	SHexCer
Sphingolipids—Phosphosphingolipids—Ceramide phosphocholines (sphingomyelins)	SM
Sphingolipids—Sphingoid bases	SPB
Sterol Lipids	ST
Glycerolipids—Triradylglycerols—Triacylglycerols	TG
Fatty Acyls—Fatty esters—Wax esters and diesters	WE

### Identifying significant lipids and lipid categories- Tiers 1 and 2

**Fig 6:**
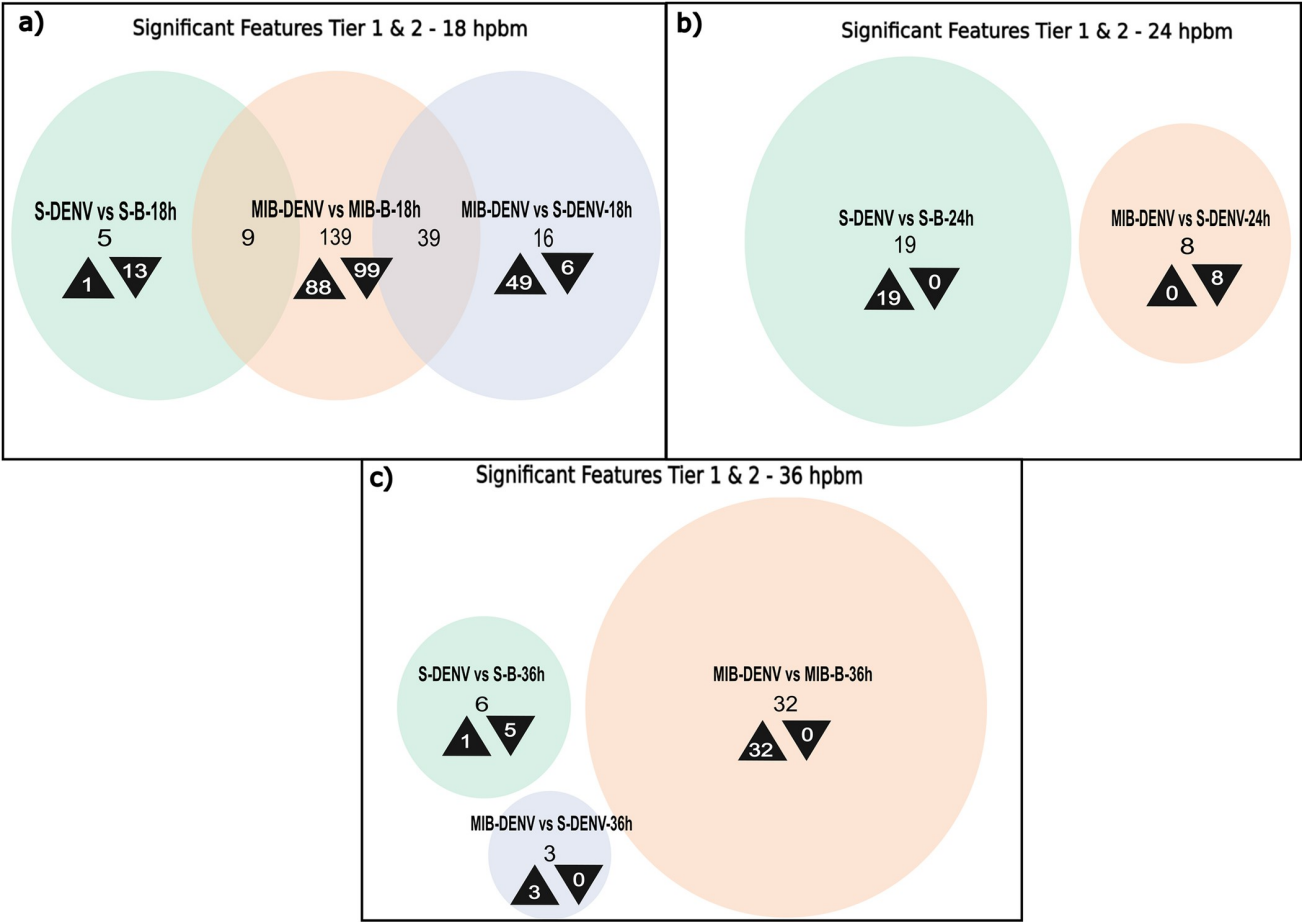
Venn Diagram of significant features, Tier 1 and 2–18, 24, 36 hpbm. Venn Diagrams showing the number of significantly differently regulated metabolites selected using the SAM model at each time point for each sample comparison (Cali-S+DENV vs. Cali-S+B, Cali-MIB+DENV vs. Cali-MIB+B, and Cali-MIB+DENV vs. Cali-S+DENV). Black arrowheads pointing up show the number of metabolites that were increased in concentration compared to the control group, and arrowheads pointing down show the number of metabolites that were decreased in concentration compared to the control. The control groups are listed second in each comparison.

Using the lipids identified in tiers one and two, 14, 187, and 56 lipids were significant different at 18 hpbm in Cali-S+DENV vs Cali-S+B control, Cali-MIB+DENV vs Cali-MIB+B controls, and Cali-MIB+DENV vs Cali-S+DENV, respectively (Fig 6A). Many lipids with significantly different concentrations were shared among the Cali-MIB comparisons (39 lipid features). Cali-S+DENV midguts vs Cali-S+B controls had primarily downregulated features (93%). Whereas Cali-MIB+DENV vs Cali-S+DENV had primarily up-regulated features (89%) (Fig 6A). Cali-MIB+DENV vs Cali-MIB+B controls had an approximately even distribution of upregulated to down-regulated lipids (47% upregulated) (Fig 6A).

At 24 hpbm, Cali-S+DENV midguts had 19 significant lipids when compared with Cali-S+B controls, and all lipids were upregulated (Fig 6B). Cali-MIB+DENV vs Cali-S+DENV samples had eight features achieve statistical significance, and all showed reduced concentrations in the Cali-MIB strain. The Cali-MIB+DENV vs Cali-MIB+B comparison had no significant features at the 24 hpbm time point (Fig 6B). The 36 hpbm time point had 6, 32 and 3 significant features for the Cali-S+DENV vs Cali-S+B, Cali-MIB+DENV vs Cali-MIB+B, and the Cali-MIB vs Cali-S comparisons, respectively (Fig 6C). Interestingly, none of the features were common among these groups. Both Cali-MIB+DENV vs Cali-MIB+B and Cali-MIB+DENV vs Cali-S+DENV had entirely upregulated significant features, whereas the Cali-S+DENV vs Cali-S+B comparison had primarily decreased concentrations of lipids being significant (83%) (Fig 6C). The identities of the top 10 significantly changed features for each comparison described above are presented in Table 3.

**Table 3:** Top 10 significantly identified features in tiers 1, and 2. Significance was determined using SAM modelling, with an empirical FDR at 10%. If less than ten features were significant at the 10% threshold, then only those features are listed. Features are listed in alphabetical order, with features appearing more than once included in bold typeface.

**18 HPBM**		
Cali-S-DENV vs Cali-S-B 18hpbm	Cali-MIB-DENV vs Cali-MIB-B 18hpbm	Cali-MIB-DENV vs Cali-S-DENV 18hpbm
PE O-18:1_22:6-DR	Cer d15:0_20:0;O2-DR	Cer d16:0_16:0;O2-UR
FA 22:6-DR	Cer d17:0_20:0;O2-DR	**Cer d18:0_18:0-UR**
LDGTS 24:4-DR	**Cer d18:0_18:0-UR**	**Cer d18:0_22:0;O2-UR**
PC 16:0_20:3-DR	**Cer d18:0_22:0;O2-UR**	**Cer d18:0_22:1;O2-UR**
PC 16:4_24:4-DR	**Cer d18:0_22:1;O2-UR**	**Cer d18:1_18:1;O3-UR**
PE O-16:1_22:4-DR	**Cer d18:1_18:1;O3-UR**	**Cer d22:0_16:0;O2-UR**
PE O-18:1_22:4-DR	**Cer d22:0_16:0;O2-UR**	DG 6:0_27:0-UR
SM d14:0_22:0-DR	**MG 17:0-UR**	**MG 17:0-UR**
SM d14:2_17:0-UR	**TG 16:1_18:1_20:3-DR**	PE 16:4_24:4-UR
**TG 12:0_22:2_22:6-DR**	**TG 18:1_18:2_18:3-DR**	Stachybocin B (Fungi metabolite)-UR
**24 HPBM**		
Cali-S-DENV vs Cali-S-B 24hpbm	Cali-MIB-DENV vs Cali-MIB-B 24hpbm	Cali-MIB-DENV vs Cali-S-DENV 24hpbm
**PC 14:1_24:2-UR**		FA 18:0-DR
**PC 16:2_24:4-UR**		FA 20:0-DR
**PC 18:1_18:2-UR**		**PC 14:1_24:2-DR**
PC O-14:0_24:4-UR		**PC 16:2_24:4-DR**
PC O-18:0_20:4-UR		**PC 18:1_18:2-DR**
**TG 12:0_12:1_18:5-UR**		**TG 12:0_12:1_18:5-DR**
**TG 12:0_22:2_22:6-UR**		**TG 16:1_18:1_20:3-DR**
TG 16:0_20:4_22:5-UR		**TG 18:1_18:2_18:3-DR**
**TG 16:1_18:1_20:3-UR**		
**TG 18:1_18:2_18:3-UR**		
**36 HPBM**		
Cali-S-DENV vs Cali-S-B 36hpbm	Cali-MIB-DENV vs Cali-MIB-B 36hpbm	Cali-MIB-DENV vs Cali-S-DENV 36hpbm
Cer d14:2_20:0;O2-UR	PE O-18:0_16:1-UR	PR 20:0;O2-UR
DG 16:0_16:1-DR	Cer d14:2_20:1;O2-UR	Cer d16:0_22:1;O2-UR
PC 18:2_18:2-DR	Cer d18:1_24:2;O2-UR	**Cer d18:0_22:1;O2-UR**
PE 18:1_19:0-DR	CL 16:0_16:1_16:1_16:1-UR	
PEtOH 18:2_20:5-DR	HexCer d14:0_26:1;O2-UR	
SM d14:0_28:2-DR	HexCer d14:1_28:2;O2-UR	
	HexCer d18:1_24:1;O2-UR	
	PC 16:0_18:1-UR	
	PE 18:1_18:2;O-UR	
	SM d14:0_15:0-UR	

**Fig 7:**
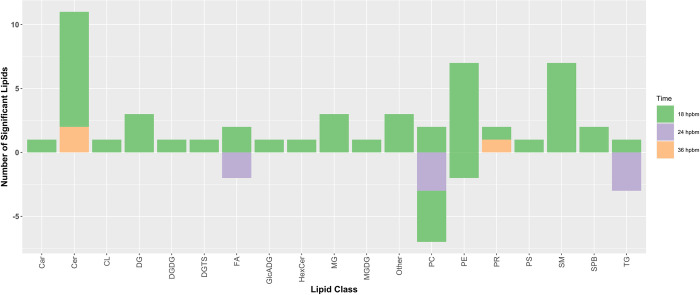
Significant Lipids by Category- Tiers 1 and 2. Number of significant lipids regulated in the Cali-MIB+DENV vs Cali-S+DENV comparison, by lipid category. Each lipid category is divided by timepoint, with 18 hpbm shown in green, 24 hpbm shown in purple, and 36 hpbm shown in orange. The number of significant lipids differently regulated is shown on the Y-axis, with positive values indicating an increase in concentration compared with Cali-S+DENV controls, and negative values indicating a decrease in concentration. Abbreviations for lipid categories shown on the X-axis can be seen in Table 2.

Lipid classes for features identified as significant in the Cali-MIB+DENV vs Cali-S+DENV comparison can be seen in Fig 7. At 18 hpbm -phosphatidylethanolamines (PE), ceramides (Cer), and sphingomyelins are the most common lipid categories (Fig 7). At 24 hpbm, only fatty acids (FA), phosphatidylcholines (PC), and triglycerides (TG) are altered (Fig 7). Similarly, at 36 hpbm, only ceramides and prenol Lipids (PR) are significantly changed.

### Lipidomic profiles of Cali-MIB and Cali-S Blood-fed midguts

We wanted to determine if the lipid profiles differ between Cali-MIB and Cali-S strains in response to blood meal alone. As such, we performed PCA analysis on Cali-MIB+B and Cali-S+B samples at all three timepoints to determine if the lipid profiles were different in the absence of DENV. The PCA plots can be seen in S1 Appendix (Fig A in S1 Appendix). This analysis was performed using the Tier 1, 2, and 3 data, as the primary goal was to identify broad patterns of profile difference, rather than identify the lipidomic substituents.

At 24 and 36 hpbm, PCA plots show separation between the Cali-MIB and Cali-S samples, which aligns with the PCA plots from DENV-challenged samples. However, at 18 hpbm, there is an overlap between the two profiles suggesting there may not be significant differences between the samples at this timepoint. Indeed, at 18 hpbm, SAM analysis shows only one significant feature, whereas at 24 and 36 hpbm there were 35 and 190 significant features, respectively (Fig B in S1 Appendix).

Additionally, there was little overlap in significant features that were altered in the Cali-MIB+B vs Cali-S+B comparison and the Cali-MIB+DENV vs Cali-S+DENV comparison at 18 and 24 hpbm. However, by 36 hpbm, 38.8% of features changed by blood meal (Cali-MIB+B vs Cali-S+B) were shared with features upregulated in Cali-MIB+DENV vs Cali-S+DENV. Overall, the results of the SAM analysis in the blood fed strains (Cali-MIB+B vs Cali-S+B) and the results presented in the Venn Diagrams indicate that the lipid profile of blood fed insects may be distinct from their virus fed counterparts at 18 and 24 hpbm (Fig B in S1 Appendix), but may share more common lipid features at 36 hpbm (Fig B in S1 Appendix).

## Discussion

### Altered lipid metabolism in DENV infected MIB populations

Lipid metabolism has been reported to influence DENV infection kinetics *in Ae*. *aegypti* [21,23,24,27] and has been proposed as an explanation for *Wolbachia*-induced DENV resistance [35]. We performed LC-MS/MS profiling of pooled samples of midguts of Cali-MIB and Cali-S, with or without DENV challenge, at three timepoints post-infectious blood meal: 18, 24, and 36 hpbm. Using this method, 18,749 features were detected, of which 863, 169, and 6223 were identified in tiers 1, 2, and 3, respectively. In total, 7255 features, or 38.7%, were positively or putatively Identified.

The timepoints for this experiment were chosen to elucidate the early mechanisms that govern viral success or resistance. The selection of earlier timepoints was based on our previous studies showing that significant remodelling of the transcriptome occurs by 24–48 hpbm in response to DENV and that these times post blood meal are critical for the virus to establish infection [13,18,29,37]. We hypothesized that metabolic shifts occurring during this time may be required for the resistant phenotype. However, the choice of earlier temporal measurements of the metabolome has limitations for detecting metabolic signals of viral resistance.

First, although DENV is expected to be establishing a replication cycle within the midgut cells at ~24hpbm, a small proportion of cells will likely be infected compared to the thousands of uninfected cells, and this may influence the ability to identify positive signals of viral resistance. Later timepoints may have provided a stronger metabolic signal of viral infection; however, the possible increase in signal may be balanced by the virus escaping the midgut and moving to other tissues for replication.

Second, upon blood feeding, the resident microbiome expands significantly, and remains high throughout blood meal digestion [38]. As blood meal digestion occurs throughout the selected timepoints, there will be a significant number of bacterial lipids identified in our screen. Moreover, with the methods chosen for analysis, it is impossible to discern which lipids are bacterially derived or synthesized by the mosquito host. Nonetheless, although some lipids identified here as significant may be of bacterial origin, they may negatively impact viral replication and determining their composition may be an important first step for elucidating their proposed anti-DENV properties.

A final confounder worth noting concerns the DENV supplied via bloodmeal. During the preparation of DENV supplemented bloodmeal, rabbit blood was mixed 1:1 with infected cell supernatant and supplied to insects for blood feeding; control insects were fed on rabbit blood alone. Given this supplementation protocol, there is potential for lipids present in the DENV cellular media to be detected within our analysis. Despite these caveats discussed above, the data presented here provide new directions for studying DENV-*Ae*. *aegypti* interactions.

### Candidate anti-DENV lipid features and classes

The purpose of this work was foremost to identify the lipidome changes that occur in response to DENV challenge and identify lipids and pathways that may enhance or diminish DENV replication in two sympatric strains of *Ae*. *aegypti* that are refractory or susceptible to infection. Therefore, we will focus our discussion on the Cali-MIB+DENV vs Cali-S+DENV comparisons. In Cali-MIB, these compounds may directly interfere with an aspect of viral entry into the cell, replication, or egress; they may act as precursors to anti-DENV immune responses and increase the concentration of immune mediators; or they may shift metabolism away from a pro-viral milieu and prevent viral components from being assembled. However, there may instances in our data where the change in a molecule may be the result of viral propagation in the Cali-S insects and not a condition of viral resistance in the Cali-MIB strain. Nonetheless, the aim of the present study is to identify lipids found in differing quantities between the groups, and these molecules are still of interest for further investigation.

Lipids are highly diverse macromolecules with numerous categories, classes, and subclasses. Because of the enormous diversity of lipid structures, this classification system is often used to report lipidomics results within the literature. Here, we will discuss the significant features within the lipid categorization system described in Table 2. The lipid categories discussed here have the greatest number of significantly changed features in the Cali-MIB+DENV vs Cali-S+DENV comparison. We will place an emphasis on phospholipids and ceramides due to their previously identified involvement in viral infection and apoptosis, respectively [21,27,39–43]

### Sphingolipids (SLs)

SLs are integral components of cellular membranes that are well conserved across eukaryotic organisms but also are found in a diverse range of species, including bacteria [44,45]. Recent discoveries have shown that SLs are essential components of membrane-rafts and numerous signalling pathways [45,46].

SLs are amphipathic molecules made of a sphinganine or sphingosine backbone with a fatty acid molecule attached; they comprise up to 30% of cellular membranes [45]. Ceramide makes up the basic building block of SLs and is formed through linking a long-chain fatty acid to sphingosine via an amide bond [24,45]. The most common subclasses of SLs identified as significantly altered in our analysis were ceramides, sphingomyelins (SM), and ceramide phosphoethanolamines (PE-Cer).

Ceramides are involved in numerous cellular processes and have gained interest in recent years due to their involvement in orchestrating cellular death and proliferation [24,44–47], and increased research has led to the expansion of known ceramide structures and their metabolism [47]. Perhaps the most well-studied function of ceramides and SLs is their role in regulating apoptosis and autophagy. Ceramides and sphingosine-1-phosphate function to counter each other; the former induces a state of cell death (apoptosis), and the latter can induce cell survival pathways such as autophagy [39,48]. These two compounds have been described as a molecular “switch” between these cellular states [39]. The mechanism of ceramide-induced cell death is not well resolved. However, a key step during the initiation of apoptosis involves the permeabilization of the mitochondrial membrane, and membrane permeability increases with increased ceramide concentrations [42]. Counter to ceramides, S1P has been shown to enhance pro-survival and growth pathways such as mTOR [39]. Both autophagy and apoptosis have demonstrated involvement with DENV infection. Autophagy is crucial for the establishment of DENV infection in *Ae*. *aegypti* [23,49–52], whereas apoptosis may function to limit viral replication; we have shown previously that in Cali-MIB insects, apoptosis can function to control DENV infection [15].

Additionally, ceramides and their derivatives have been shown to be important for membrane curvature and fluidity, and thus can influence sites of viral replication and packaging. Ceramide is a cone-shaped lipid that is highly hydrophobic and can induce membranes to form a negative curvature, influencing vesicle formation and trafficking [43]. Furthermore, SM is a ceramide derivative with an attached phosphorylcholine that can be converted back to ceramide through a degrative pathway [45]. SM is speculated to be important in lipid rafts, membrane fluidity, cell signalling, and other ordered membrane domains [45,53,54]. PE-Cer is a SM derivative that has shown increased importance in invertebrates [53]. In mammals, SM interacts with cholesterol to increase membrane fluidity and membrane packing to facilitate various cellular processes [53]. Although the function of PE-Cer is not well understood, it does not interact favourably with cholesterol to enhance membrane fluidity [53]. However, all the above mechanisms are dependant on the length of the ceramide FA chain, with long chain and very long chain ceramides inducing different signalling events and membrane properties [43].

The influence of ceramides on viral infection appears to vary for different viruses [43]. *Aktepe et al*. demonstrated that West Nile virus (WNV) replication was diminished upon cellular depletion of ceramides, whereas DENV replication was enhanced, indicating that higher concentrations of ceramides negatively impact DENV replication [43]. This may be due to the diverse mechanisms through which ceramides can influence metabolism. In our lipidomics analysis, ceramide compounds were overrepresented as a significantly altered lipid class and were primarily upregulated (Figs 5 and 7). Given our previous research demonstrating increased apoptosis in the midguts of Cali-MIB insects, we postulate that increases in ceramide concentrations may initiate an increased apoptotic response in the midguts of Cali-MIB mosquitoes. Previously, our group performed a transcriptome analysis of Cali-S and Cali-MIB insects after DENV challenge [17]; we utilized this data to query if there were any transcriptional changes to ceramide processing enzymes. Indeed, one sphingomyelin phosphodiesterase (AAEL006381), responsible for breaking sphingomyelin down to phosphocholine and ceramide, was significantly upregulated at 36 hpbm in the refractory strain (adjusted p-value = 0.0136) [17]. As both, our lipidomics and transcriptomics datasets identified alterations in ceramides and enzymes related to the metabolism of ceramides as significant, we consider ceramide metabolism as a potential mechanistic driver for viral resistance in the Cali-MIB strain that warrants further investigation.

In addition to changes in ceramides, numerous SMs and PE-Cer compounds were significantly upregulated during DENV infection in Cali-MIB midguts at 18 and 36 hpbm (Figs 5 and 7). Interestingly, very few changes in SM species and no changes in PE-Cer were observed at 24 hpbm; those SM species that were significantly different showed decreased concentrations in Cali-MIB samples. In contrast, no PE-ceramide species and only one SM species were identified as significant at any time point in the DENV-challenged Cali-S strain compared to its blood-fed controls, suggesting that SM and PE-Cer species may have direct or indirect anti-viral action. Other studies on lipidomics results with SM and PE-Cer regulation have found contradictory results [24,55]. *Chotiwan et al*. [24] reported decreased PE-Cer concentrations at early infection timepoints (3 days post blood meal) and no changes in SM (mosquito midguts); whereas *Perera et al*. [56] reported ~2-fold increases in SM and reduced concentrations of PE-Cer (C6/36 cells). The reasons for the discrepancies between these studies, including ours, may be related to differing tissues (midguts of refractory insects, midguts of wild-type insects, C6/36 cell culture) or differing time points.

### Glycerolipids (GLs)

Glycerolipids form a diverse category of structures with a glycerol backbone linked to at least one hydrophobic chain. They can be subclassified as nonpolar or polar glycerolipids; the former contains mono-, di, and triacylglycerols (MAGs, DAGs, TAGs), and the latter contains the phospholipids (PLs), including phosphatidic acid (PA), phosphatidylcholine (PC), phosphatidylethanolamine (PE), phosphatidylserine (PS), and phosphatidylinositol (PI).

DAGs and TAGs can be utilized for energy metabolism in the insect. After a bloodmeal, fatty acids are absorbed and can be converted to TAGs and, to a lesser extent, DAGs for energy storage [24,57–59]. Within cells, DAGs also serve as second messengers for cell signalling, regulating proliferation, mitochondrial function, and apoptosis [24]. TAGs are non-polar lipids and are often stored in lipid droplets (LD) within the cytosol [58,59]. In contrast, phospholipids are amphipathic lipid molecules that contain a polar headgroup and non-polar tails [58,59]. Phospholipids make up most cellular membranes, and the composition of phospholipids impacts membrane properties and functions [58,59].

In our study, the GLs that were most significantly dysregulated in Cali-MIB+DENV samples were DAGs, TAGs, PC, PE, and PG. These lipids are intricately related; DAG can be converted to PC or PE as membrane components, or the production of TAGs for energy storage [60,61]. Interestingly, TAGs are primarily stored within the cytoplasm in lipid droplets, which are surrounded by a monolayer of amphipathic phospholipids, mainly of PC and PE [61]. However, lipid droplets and the phospholipids PC and PE appear to have opposing roles in DENV infection [28,62] and DENV has been reported to halt the de novo synthesis of PL to aid its own replication [28]. Additionally, the induction of PL synthesis negatively influences viral particle assembly [28]. There is an accumulation of LDs in the cytoplasm after DENV infection and these may enhance the viral infection [62]. With our current data, it is not possible to ascertain whether changes in PLs influence LDs on DENV replication, and whether increased TAG concentrations correlate with LD density in the cytoplasm.

An alternative explanation of the differences in DAG and PLs between Cali-MIB and Cali-S relates to the PL remodelling. The various phospholipids can be produced through the remodelling of PC via the Lands cycle, while PC is produced *de novo* through the reaction of DAG and phosphocholine [28,63]. Subsequently, a fatty acid is removed from PC to produce lysophophatidylcholine (lysoPC), which then can be modified to give rise to the other PLs [63]. Both, synthesis of phospholipids de novo and through phospolipid remodelling regulate the amount and types of PLs produced, and hence influence membrane properties. DENV has been shown to induce phospholipid remodelling in *Ae*. *aegypti* early in infection, as noted by increases in lysophospholipids (lysoPL) [24,28] and activation of the remodelling pathway is speculated to increase PL diversity to positively influence virion formation. Similarly, PE and DAG can induce a negative curvature of the membrane [64], and perhaps their mechanism of action is similar to ceramides [43]. Our data revealed few changes in lysoPLs, but significant increases in the concentrations of PC and PE; this may indicate that in Cali-MIB, PL remodelling is perturbed in response to DENV.

### Fatty acids (FAs)

FAs contribute to numerous fundamental metabolic constituents that are precursors to structural, energetic, and signalling molecules, including many of the molecules discussed above (TAGs, DAGs, Phospholipids) [65,66]. Often, fatty acids form ester bonds with alcohol groups of other molecules, such as glycerol. Hence, we distinguish between esterified FAs which are those contained in molecules such as TAGs and DAGs, and free FAs (FFA). FFAs are used for energetic reactions and require activation to function in many metabolic pathways [65,67]. Activation involves esterification with a CoA molecule, a requirement for β-oxidation in the mitochondria [65,67]. Fatty acid esters, such as TAGs, are broken down to produce FFAs for oxidation and production of other structural and metabolic lipids. Importantly, accurate quantification of FFA is challenging because during sample preparation, the hydrolysis of lipids may unnaturally increase the concentration of FFA.

There are numerous studies showing perturbed FA metabolism in DENV-infected tissues and that influencing the production of FAs through modulating fatty acid synthase affects DENV replication [55,68,69]. However, it appears that the overall concentration of FA is not a determinant of a pro-DENV milieu but rather the ratio of activated FA to FFA [24]. While studying the anti-DENV effects of *Wolbachia* infection, it was noted that *Wolbachia* co-infection reduces concentrations of activated FA and increases FFA concentrations. Interestingly, attempts to determine the effect of FFA on DENV replication through dsRNA knockdown of a lipolytic enzyme (pancreatic lipase-related protein 2) that produces FFA from TAGs failed to observe changes in DENV replication [25], possibly because FFAs can be obtained from numerous sources.

Perhaps the most salient feature of FA in the context of immune mechanisms is their function as signalling molecules. Eicosanoids are derivatives of 20 carbon polyunsaturated FAs (PUFAs) that are recognized for their role in mediating immune reactions in vertebrates and invertebrates [70–73]. One class of eicosanoids, the prostaglandins (PGs), has been directly implicated in immune responses to various pathogens in insects [72,74]. PGs are synthesized from the 20 carbon arachidonic acid, which can be derived from the essential fatty acid linoleic acid (18:2) [72]. However, it is important to note that the importance of PGs in viral infections in insects has not been determined [75]. In our study, numerous FAs were altered in the Cali-MIB strain challenged with DENV compared to the Cali-S strain challenged with DENV (Figs 5 and 7). FAs were primarily upregulated, except for the 24 hpbm timepoint. FAs with significant concentration changes were primarily long-chain FAs (13–22 carbons in length) or very-long-chain FAs (22+ carbons) [67,76]. Moreover, no activated FAs, or fatty Acyl CoAs, were significantly different in Cali-MIB midguts. Among these changes in FAs, many C18 and C20 PUFAs were upregulated in the Cali-MIB midguts compared to Cali-S, possibly indicating increased lipid mediator production for that may be involved in immune activation.

### Sterol lipids (STs)

Sterols are ringed lipid alcohols present in almost all living organisms, from bacteria to vertebrates [77,78]. They are involved in eukaryotic membrane function, regulating metabolism, and developmental signalling [79,80]. Although sterols perform very similar functions in vertebrates and invertebrates, insects lack the ability to synthesize sterols *de novo* and must obtain them from their diets [79]. As in vertebrates, cholesterol is the dominant sterol found in invertebrates [79].

Our data show that sterols were significantly altered in response to DENV challenge in Cali-MIB insects when compared with Cali-S insects challenged with DENV (Fig 5). Interestingly, there appeared to be temporal regulation of sterols. At 18 and 36 hpbm, sterols were primarily upregulated, whereas, at 24 hpbm, all significant sterols were down-regulated (Fig 5). Many of the sterol lipids regulated early in infection (18 hpbm) were cholesterol and cholesterol derivatives. In contrast, those shown to be significant at later time points had diverse sterol categories.

Numerous articles have reported the effects of cholesterol on flavivirus replication in mammals and *Ae*. *aegypti* [19,20,22,81–83]. Cholesterol is essential for DENV entry, and selective depletion of cholesterol hinders viral success [22,83]. Moreover, to facilitate viral replication, flaviviruses have demonstrated the ability to influence cellular concentrations of cholesterol [19,82]. It is theorized that cholesterol competition is one of the mechanisms of viral resistance among *Wolbachia* transinfected insects [20,25,26,81]. However, the relationship between cholesterol and viral success may depend on cholesterol concentration and timing. Viral entry and uncoating early in infection may be negatively influenced by high cholesterol concentrations [22]. Additionally, higher cholesterol levels are associated with DENV blocking, and importantly, esterified cholesterol and not free cholesterol are correlated with DENV blocking [81].

### Lipid metabolism changes in response to bloodmeal

We analyzed the data to determine if the lipidomic changes in the Cali-MIB strain were a response to the virus or a conserved response to the blood meal. We observed the metabolic differences in Cali-MIB and Cali-S strains in response to blood meal at all three timepoints (S1 Appendix). The results showed at 24 and 36 hpbm, the lipidomic profiles of Cali-MIB and Cali-S strains differed in response to blood meal, but at 18 hpbm, the profiles were similar. Moreover, in Cali-MIB samples at the 36 hpbm timepoint, many of the changed lipid features were similarly altered in Cali-MIB midguts after DENV challenge. Additionally, many of the categories for significant lipids were similar to those found in the Cali-MIB+DENV screen.

Taken together, these results indicate that lipid metabolism in response to bloodmeal is altered in the Cali-MIB strain compared to Cali-S samples, and that there are consistent features upregulated by blood meal and blood meal with DENV at 36 hpbm. Perhaps the features identified at 36 hpbm in the bloodmeal with DENV screen are less involved in maintaining DENV resistance and more indicative of metabolic demands associated with blood meal digestion.

## Conclusions

We present the first lipidomics profiling of sympatric DENV-refractory and-susceptible strains of *Ae*. *aegypti* challenged with DENV. In lipidomics analyses done to date, only susceptible populations of *Ae*. *aegypti* have been studied, and thus, the data cannot explain metabolic changes and immune responses that related to infection. Therefore, our experimental design offers considerable advantages as the metabolic milieu present in refractory insects is more likely to represent one that negatively impacts viral infection, replication, and transmission.

The lipidome is extremely diverse, understudied, and the literature available for individual lipid moieties is inadequate to determine their role in DENV replication. Thus, the resolution of our data must be considered when connecting lipidomics data to a phenotypic difference. If we attempt to describe lipidomic changes with high granularity, we are more likely to make erroneous claims regarding the significance of our findings. This final point highlights the fact that our data are correlative, and none of the compounds identified here as significant have been validated to be causally important for DENV resistance in Cali-MIB insects.

Notwithstanding these limitations, we have identified numerous lipid compounds and categories that may possess anti-DENV character, such as sphingolipids and ceramides, glycerolipids and phospholipids, fatty acids, and sterols. Perhaps the most intriguing of our results is the rapid increase in ceramide compounds within the Cali-MIB refractory population, as ceramides have demonstrated ability to initiate apoptosis [39,48]. The stark increase in ceramide compounds seen in our analysis, coupled with our previous work demonstrating a rapid onset of apoptosis in the refractory Cali-MIB strain, provides a possible mechanism for apoptotic viral resistance in Cali-MIB females. This hypothesis should be evaluated using traditional molecular approaches to determine the magnitude of ceramides’ effect on DENV replication. Moreover, there may be a reduced induction of phospholipid remodelling in Cali-MIB midguts, indicated by increased concentrations of PC and PE, with minimal perturbations in lysoPLs. To evaluate if lysoPL remodelling is stunted in refractory Cali-MIB insects, concentrations of lysoPLs and PLs could be modified, and the impacts on DENV replication observed, as has been done previously [28].

In addition to the contributions to our understanding of DENV-vector interactions, the data produced by our lipidomics analysis provide a rich source of information that will be highly valuable to other researchers interested in understanding regulatory compounds, such as lipid mediators of inflammation, or mechanisms of membrane function. The LC-MS data has been made publicly available for reproduction and for individuals to investigate how their area of research may be influenced by DENV infection. Overall, this research establishes numerous hypotheses to be systematically tested to validate novel anti-DENV lipid pathways in mosquito vectors and significantly advances our understanding of the intricate and intimate molecular interactions between vectors and pathogens that determine or govern DENV transmission.

## Supporting information

S1 Appendix Lipidomics Analysis of Blood-Fed Cali-MIB and Cali-S Midguts. **Fig A** Tier 1, 2, 3: PCA results of Cali-MIB and Cali-S samples fed on blood only. **Fig B** Venn Diagram of significant features in Cali-MIB vs Cali-S fed on blood only, Tier 1 and 2–18, 24, and 36 hpbm. **Fig C** Significant lipids by category-Tiers 1, 2, and 3. Number of significant lipids regulated in the Cali-MIB+blood vs Cali-S+blood comparison, by lipid category.
(PDF)

S1 Table List of identified and normalized features from lipidomics LC-MS/MS. (XLSX)

S2 Table List of unidentified features from lipidomics LC-MS/MS. (XLSX)

S3 Table List of features identified as significant by SAM, Tiers 1, 2, and 3. (XLSX)

S4 Table List of features identified as significant by SAM, Tiers 1, and 2. (XLSX)
