# Supplementary material for: Profiling lipidomic changes in dengue-resistant and dengue-susceptible strains of Colombian Aedes aegypti after dengue virus challenge
Source: PLoS Negl Trop Dis. 2023 Oct 17;17(10):e0011676. doi: 10.1371/journal.pntd.0011676 (PMC10581493; doi:10.1371/journal.pntd.0011676)
Supplement: S1 Appendix — Fig A Tier 1, 2, 3: PCA results of Cali-MIB and Cali-S samples fed on blood only. Fig B Venn Diagram of significant features in Cali-MIB vs Cali-S fed on blood only, Tier 1 and 2–18, 24, and 36 hpbm. Fig C Significant lipids by category-Tiers 1, 2, and 3. Number of significant lipids regulated in the Cali-MIB+blood vs Cali-S+blood comparison, by lipid category. (PDF) [file pntd.0011676.s001.pdf]

S1 Appendix: Lipidomics Analysis of Blood-Fed Cali-MIB and Cali-S Midguts

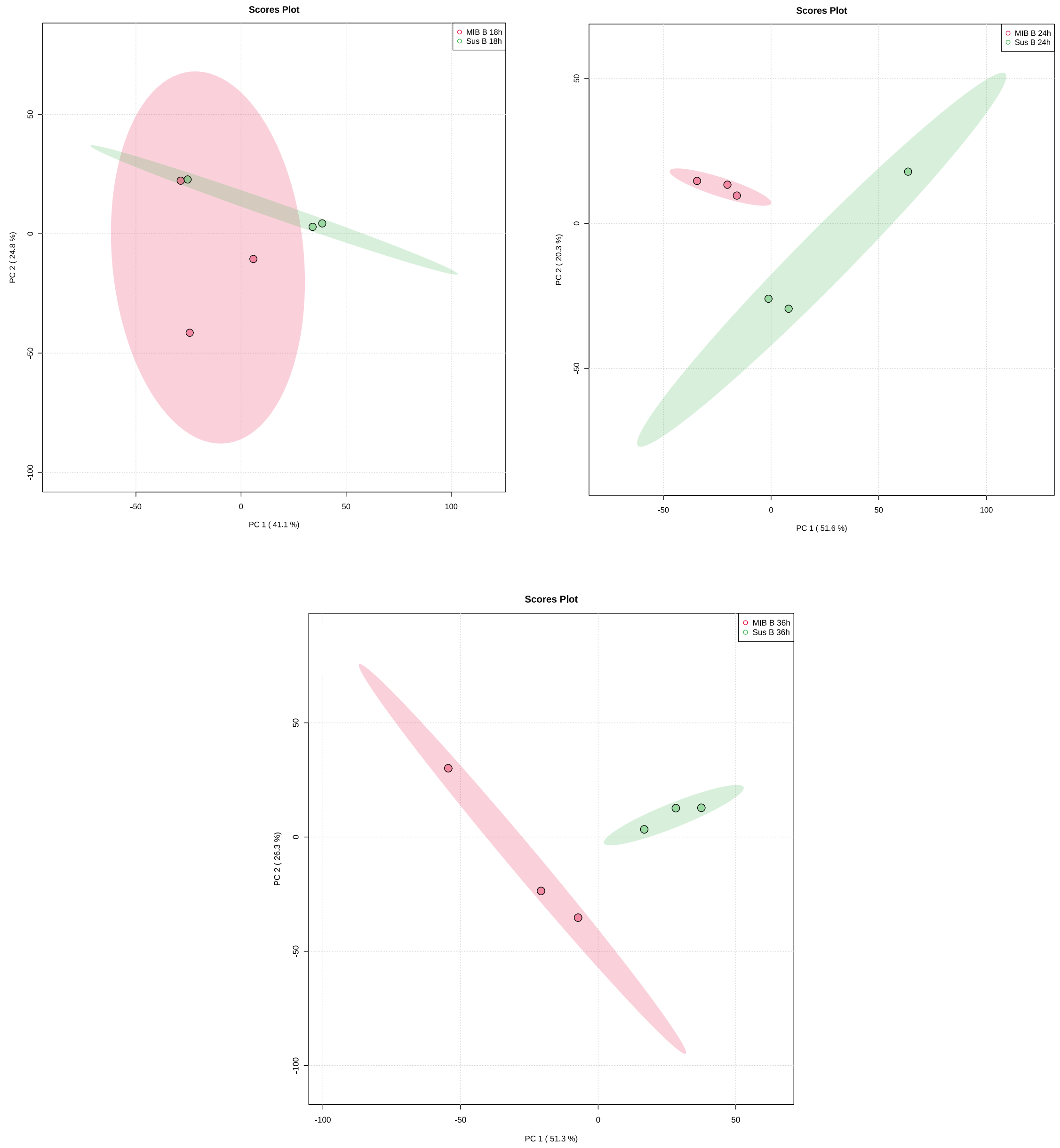

Fig A **Tiers 1, 2, and 3: PCA plots of Cali-MIB+Blood and Cali-S+Blood lipidomics profiles. Principal component analysis (PCA) of Cali-MIB+Blood vs Cali-S+Blood, 18 hpbm, Cali-MIB+Blood vs Cali-S+Blood, 24 hpbm, and Cali-MIB+Blood vs Cali-S+Blood, 36 hpbm. The X-axis has the first principal component and the Y-axis the second. Each data point represents one sample of 10 pooled insect midguts with the 95% confidence interval displayed as the ellipsis.**

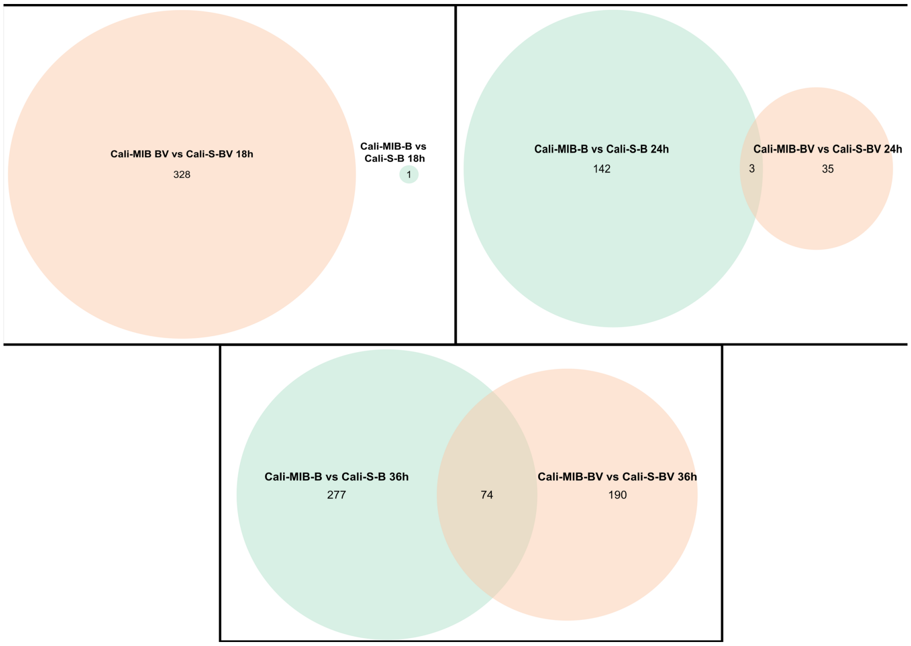

Fig B **Venn Diagram of significant features, Tier 1, 2, and 3- 18, 24, and 36 hpbm. Venn Diagrams showing the number of significantly differently regulated lipids selected using the SAM model at each time point, for each sample comparison (Cali-MIB+Blood vs Cali-S+Blood, Cali-MIB Blood+Virus vs Cali-S Blood+virus). The control groups are listed second in each comparison.**

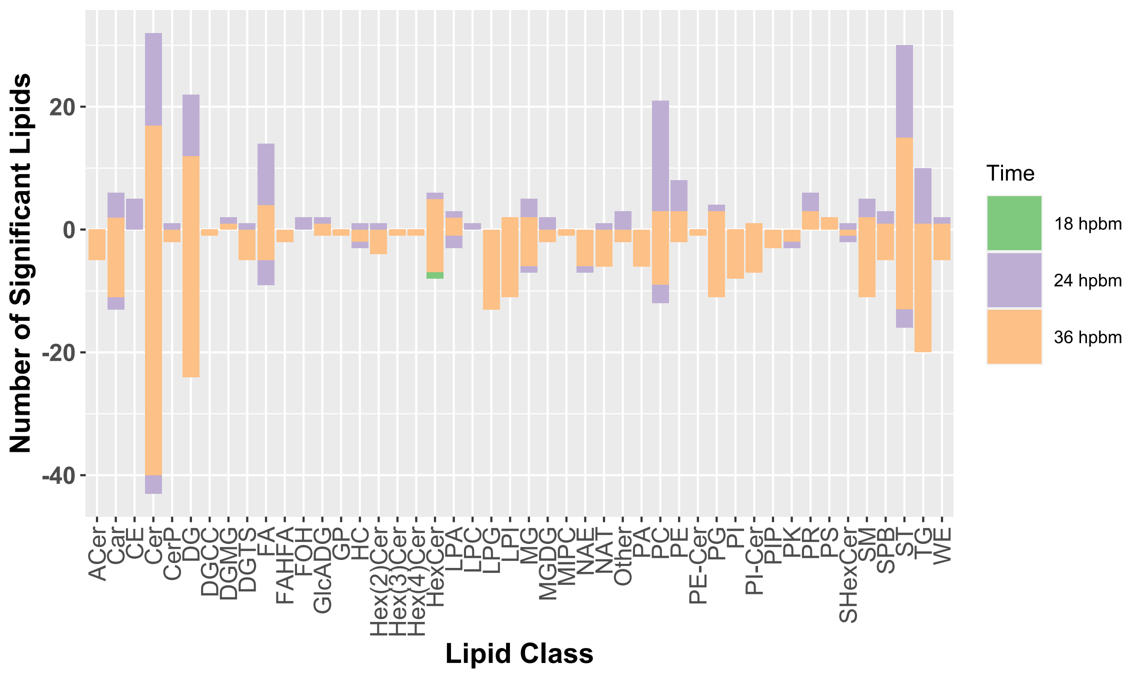

Fig C **Significant lipids by category-Tiers 1, 2, and 3. Number of significant lipids regulated in the Cali-MIB+blood vs Cali-S+blood comparison, by lipid category. Each lipid category is divided by timepoint, with 18 hpbm shown in green, 24 hpbm shown in purple, and 36 hpbm shown in orange. The number of significant lipids differently regulated is shown on the Y-axis, with positive values indicating an increase in concentration compared to control, and negative values indicating a decrease in concentration. Abbreviations for lipid categories shown on the X-axis can be seen in Table 2.**
